# Supplementary material for: Aggregative Swab Sampling Method for Romaine Lettuce Show Similar Quality and Safety Indicators and Microbial Profiles Compared to Composite Produce Leaf Samples in a Pilot Study
Source: Foods. 2024 Sep 27;13(19):3080. doi: 10.3390/foods13193080 (PMC11476302; doi:10.3390/foods13193080)
Supplement: Supplementary file 1 [file foods-13-03080-s001.zip › foods-3192260-supplementary.pdf]

Supplemental Material

Storage decreased APC counts in swabs and produce leaves. Storage decreased the aerobic plate counts in swab sample and produce leaf PBS extracts. The PBS wash from the swabs and field produce leaf samples underwent a three-month storage at 4°C for logistical reasons before DNA was extracted. The APCs were repeated before DNA extraction to measure the number of viable cells in which DNA was being extracted from. Both swab sample and produce leaf sample types regardless of inoculation status showed a decrease in aerobic plate counts following storage. Mean APC of swabs before storage was 9.21 log(CFU/g) and dropped almost one log for a mean of 8.24 log(CFU/g). Mean APC of produce leaves before storage was 9.17 log(CFU/g) and dropped more than one log for a mean of 7.70 log(CFU/g) after storage (Supplemental Figure S2). A three-way ANOVA was done to test the effects of storage, sample type, and inoculation status. Inoculation and all interaction terms were not significant (p>0.05). Storage and sample type were significant (p<0.05). Upon further investigation, sample type was not significant by a post-hoc Tukey HSD test (p=0.221). However, a post-hoc Tukey HSD test confirmed that storage was in fact significant (p<0.05).

Swabs and produce leaves had some of the most abundant taxa in common. The bacterial communities of field swabs and produce leaves were dominated by the *Alphaproteobacteria* class (19.2% and 20.3% on average, respectively) and *Gammaproteobacteria* class (71.9% and 46.6%, respectively) which both belong to the *Proteobacteria* phylum, the most abundant phylum present. Other most abundant taxonomic classes shared by swabs and produce leaf samples were *Bacilli* (0.9% and 6.4%, respectively) and *Bacteroidia* (4.1% and 14.4%, respectively). Most abundant classes that differed between the field sample types were *Verrucomincrobiae* in swabs (3.6%) and *Actinobacteria* in produce leaves (9.0%). Overall, swabs and produce leaves shared 11 classes,

produce leaves had 13 unique classes not in swabs, and swabs had 1 class not in produce leaves. The swabs and field produce leaf samples appear to be more similar to each other compared to the store produce leaf outgroup which had a lower percentage of *Alphaproteobacteria* (0.3%), absence of the *Bacteroidia* class from the top five classes, a higher percentage of *Gammaproteobacteria* (84.8%), plus the addition of *Cyanobacteriia* (11.0%) which was not in the five most abundant classes for either swabs nor produce leaves (Figure 5b).

The most abundant orders shared by field swabs and produce leaf samples were *Burkholderiales* (11.9% and 13.7% on average, respectively), *Pseudomonadales* (36.3% and 26.3%, respectively), and *Rhizobiales* (8.0% and 10.3%, respectively). Orders not in the five most abundant in swabs and produce leaves fell into the "other" category (13.9% and 34.0%, respectively). Most abundant orders that were different between sample types were *Caulobacterales* (8.7%) and *Enterobacterales* (21.3%) in swabs and *Flavobacteriales* (7.3%) and *Micrococcales* (8.5%) in produce leaves. In swabs, it was once again seen that they had a higher percentage of *Gammaproteobacteria* with swabs having two orders in the top five belonging to the *Gammaproteobacteria* class (*Enterobacterales* (21.3%) and *Pseudomonadales* (36.3%)), while produce leaves only had one order in the top five belonging to the *Gammaproteobacteria* class (*Pseudomonadales* (26.3%)). Interestingly, while swabs and produce leaves had very similar percentages of the *Alphaproteobacteria* class, when looking closer at the order rank swabs had a higher percentage of bacteria belonging to the *Alphaproteobacteria* class. For example, swabs contained both *Cualobacterales* (8.7%) and *Rhizobiales* (8.0%). Meanwhile produce leaves had a similar abundance of *Rhizobiales* (10.3%), but not enough *Caulobacterales* to make it in the top five most abundant category. Overall, swabs and produce leaves shared 32 orders, produce leaves had 25 orders not in swabs, and swabs had 11 unique orders not in produce leaves. The store

produce leaf outgroup appeared not as similar as the field swabs and produce leaves. The store produce leaf group was less diverse with nearly all the orders present belonging to only five orders ("other" category was 0.7%) and most bacteria present belonging to the *Pseudomonadales* order (80.0%) (Figure 5c).

Supplemental Figures

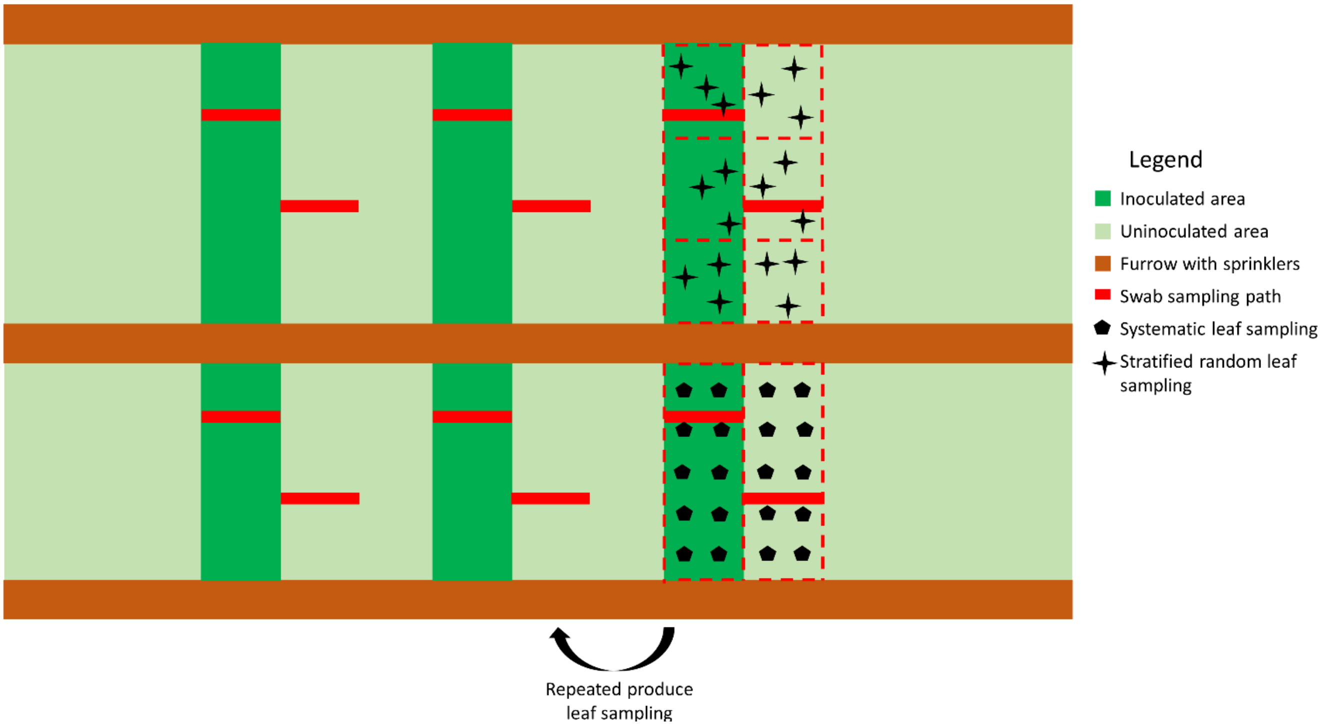

Supplemental Figure S1. A diagram representing the field with three inoculated subsections, three uninoculated subsections, and swabs and produce leaf samples taken from all subsections. The field is not drawn to scale. The systematic and stratified sampling points, and swab sample paths, represent the patterns and relative locations for sampling, not the exact number or locations within the field. Although only one type of sampling is shown on a given zone, paired systematic and stratified samples were collected from each inoculated and uninoculated zone,

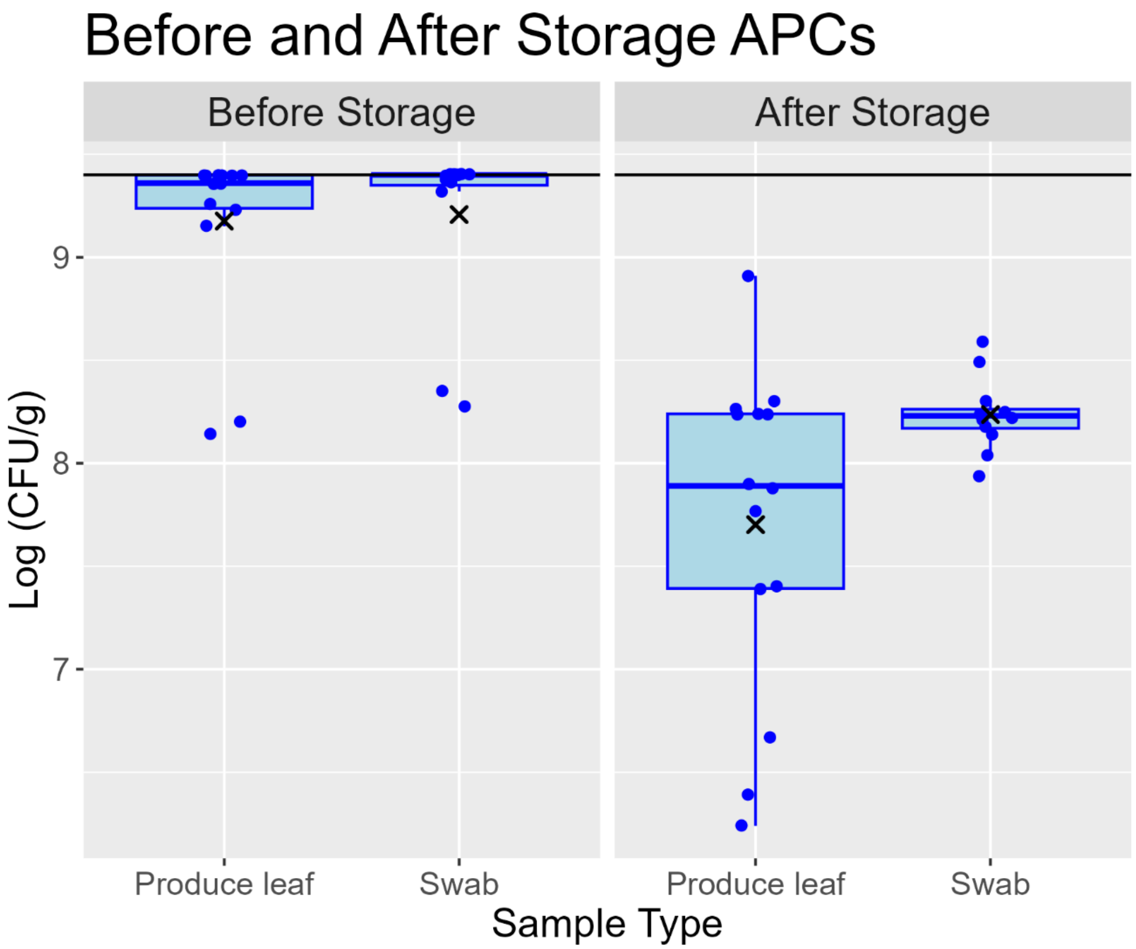

Supplemental Figure S2. Boxplots comparing aerobic plate counts from swabs and produce leaves before and after storage. Solid black line represents the upper limit of quantification of 9.40 log(CFU/g). For swabs, CFU/g refers to CFU per gram of matter picked up by the swabs. Data points are jittered so all points are visible; therefore, some points may appear slightly above limit of quantification. The middle lines of the boxplots are the medians, and the "╳" are the means. The lower and upper hinges represent the 25th and 75th percentiles. Whiskers extend to the largest or smallest values that are not beyond 1.5*IQR from the hinge. Data points beyond the whiskers are plotted individually as outliers.

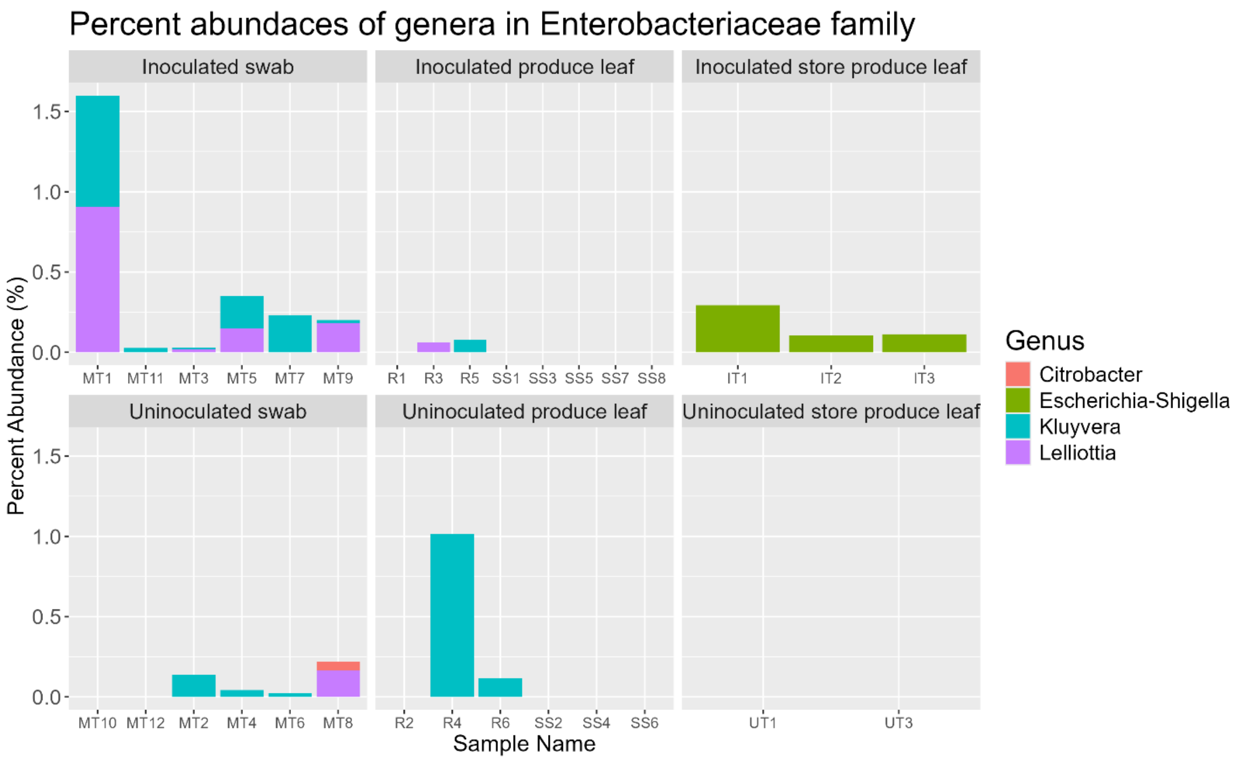

Supplemental Figure S3. Bar plot of the percent abundances of the genera that belong to the *Enterobacteriaceae* family separated by sample type and inoculation status. Nonrarefied data was used.

72

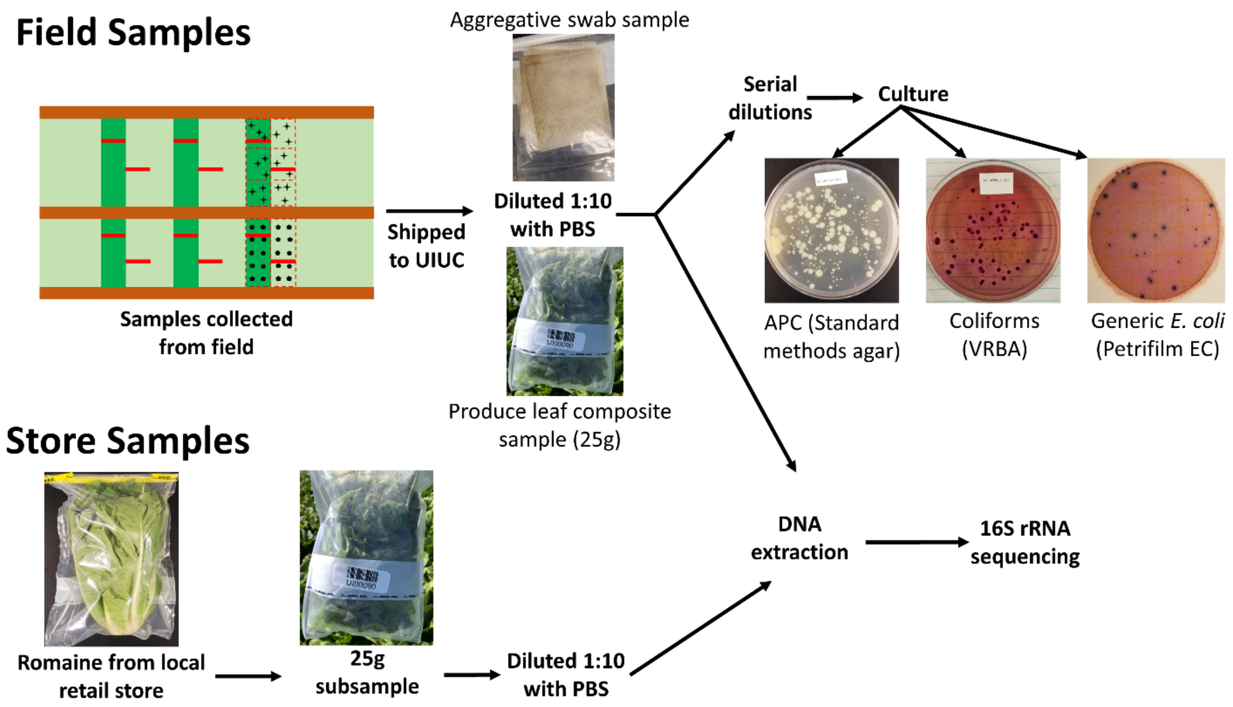

73 **Supplemental Figure S4.** A schematic of the laboratory methods preformed for samples collected
74 from the field and samples bought from a local retail store which were used as an outgroup.
